# High Vitamin D Consumption Is Inversely Associated with Cardiovascular Disease Risk in an Urban Mexican Population

**DOI:** 10.1371/journal.pone.0166869

**Published:** 2016-11-28

**Authors:** Paloma Muñoz-Aguirre, Edgar Denova-Gutiérrez, Mario Flores, Eduardo Salazar-Martínez, Jorge Salmerón

**Affiliations:** 1 Unidad de Investigación Epidemiológica y en Servicios de Salud, Instituto Mexicano del Seguro Social, Cuernavaca, Morelos, México; 2 Unidad de Investigación en Epidemiología Clínica, Hospital Infantil de México “Federico Gómez”, Ciudad de México, México; 3 Centro de Investigación en Nutrición y Salud, Instituto Nacional de Salud Pública, Cuernavaca, Morelos, México; 4 Centro de Investigación en Salud Poblacional, Instituto Nacional de Salud Pública, Cuernavaca, Morelos, México; Weill Cornell Medical College Qatar, QATAR

## Abstract

**Background:**

Vitamin D deficiency is a major global public health problem. Recent epidemiological studies have assessed the relationship between vitamin D and multiple outcomes, including cardiovascular disease. However, this evidence is limited and inconclusive. Our purpose in this study was to evaluate the association between dietary vitamin D intake and cardiovascular disease risk in adult Mexican population.

**Methods:**

We conducted a cross-sectional analysis with the baseline data from 6294 men and women aged 20–80 years participating in the Health Workers Cohort Study. Data on sociodemographic, lifestyle, and medical history factors were collected with a self-administered questionnaire. Dietary intake was evaluated by using a semi-quantitative food-frequency questionnaire. Cardiovascular disease risk was calculated using a recalibration of the Framingham heart disease prediction score. To evaluate the association between vitamin D intake and 10-year cardiovascular disease risk, odds ratios (OR) and 95% confidence intervals (95% CI) were calculated using multiple logistic regression analysis.

**Results:**

A total of 6294 subjects (1820 men and 4474 women) with a mean age of 42 years, were included. Of these, subjects in the highest quintile of vitamin D intake presented lower levels of triglycerides 14.6 mg/dL (*P* for trend = 0.001); 2.0 cm less in waist circumference (*P* for trend = 0.001) and 0.8 points less in the Framingham cardiovascular disease risk score (*P* for trend = 0.002) compared with the subjects in the lower quintile of vitamin D intake. Additionally, participants in the highest quintile of vitamin D consumption were less likely to develop elevated 10-year cardiovascular disease risk, compared with those in the lowest quintile (OR = 0.51; 95%CI: 0.33, 0.77; *P* for trend = 0.007).

**Conclusion:**

Our data suggest that higher consumption of vitamin D is associated with a reduced risk of cardiovascular disease in Mexican population.

## Introduction

Vitamin D (VD) is a prohormone whose main function is to regulate calcium and phosphorus metabolism related to preserve bone mass [1]. Due to the fact that VD has a wide distribution in the body tissues, other important functions have been discovered, such as the modulation of cell growth, immune function and reduction of inflammation [2,3].

In humans, VD is primarily obtained through sun exposure, because very few foods naturally contain vitamin D, diet and dietary supplements are other sources of VD; consequently, the assessment of dietary intake of VD is valuable for nutritional epidemiological studies [4]. Vitamin D deficiency has been identified as a public health issue [4]. Whereas, in Mexican adult population, the prevalence of deficiency in 2006 was 2.0% (serum 25-OH-D <30nmol/L -<12ng/mL-) and 9.8% had serum 25-OH-D levels <50 nmol/L-<20 ng/mL [5,6]. This condition could be attributed to decreased sun exposure, scarce dietary sources of vitamin D and an increased prevalence of obesity [7].

Cardiovascular disease (CVD) is the leading cause of death and a major cause of disability worldwide [8], and the main cause of mortality in Mexican adults [9]. Epidemiological studies have suggested that lifestyle, including diet, significantly influences CVD occurrence [10]. Previous research [4, 11–13] has suggested a possible relationship between VD, CVD, and other chronic diseases. In this sense, a recent clinical trial conducted by Zitterman et al. have documented the effect of daily vitamin D supplements on CVD markers and they found that a daily vitamin D supplement (83 μg/day) is able to improve several cardiovascular disease risk markers such as triglycerides, inflammation marker tumor necrosis factor-α, endothelial function and cardiac function in overweight subjects with inadequate vitamin D status [14]. Nevertheless, the effect of vitamin D on CVD is still unclear [15].

There are some plausible mechanisms that explain how vitamin D might modify cardiovascular outcomes, vitamin D regulates the renin-angiotensin system [16], suppresses proliferation of vascular cell smooth muscle [17], ameliorates insulin resistance [18] improves endothelial cell-dependent vasodilation [19, 20], inhibits anticoagulant activity [21] and myocardial cell hypertrophy [22, 23], and may modulate macrophage activity [24] and cytokine generation [25, 26].

There are still some questions on this issue, we do not know if optimal vitamin D consumption and concentrations are a cause or a consequence of good health. Therefore, we examined the cross-sectional relation between dietary vitamin D intake and risk of cardiovascular disease as well as cardiometabolic risk factors in subjects participating in the Health Workers Cohort Study (HWCS), in Mexico. We hypothesized that high intake of vitamin D would be negatively associated with cardiovascular disease risk, independently of demographic and lifestyle factors.

## Materials and Methods

### Study population

We conducted a cross-sectional analysis of data from adults participating in the baseline assessment of the Health Workers Cohort Study (HWCS). The participants in this cohort study are middle- to low-income status, residing in urban central Mexico. The study design, methodology, and participants’ characteristics have been detailed elsewhere [27–30]. Concisely, the HWCS focuses on lifestyle and chronic disease. Participants completed baseline questionnaires providing information about demographic, behavioral, and lifestyle factors; medical history including medication use; use of multivitamins and other supplements; and anthropometric measurements and clinical evaluations were performed.

For the present analysis we included 6294 subjects aged 20–80 who provided fasting blood samples (≥ 12 hours since last meal), and were not previously diagnosed with type 2 diabetes (assessed by self-report or with levels of glucose ≥ 126 mg/dL) (n = 491) or taking lipid lowering medication (n = 567). We also excluded participants who left 10 percent or more food items blank on the questionnaire (n = 635), and who did not consume between 600 kcal and 7000 kcal daily (n = 320).

This study was planned and conducted according to the guidelines laid down in the Declaration of Helsinki. All participating institutions’ research ethics committees: Comité de Ética en Investigación, Instituto Mexicano del Seguro Social (No. 12CEI 09 006 14); Comité de ética en Investigación, Instituto Nacional de Salud Pública (No. 13CEI 17 007 36); Comité de Ética, Centro de Investigación en Ciencias Médicas (No. 1233008X0236), revised and approved the study protocol and informed consent forms. Written informed consent was obtained from each participant.

### Dietary and vitamin D intake assessment

Dietary intake data and VD consumption were collected using a 116-item semi-quantitative food frequency questionnaire (FFQ), previously validated in an adult Mexican population [31].

Vitamin D and other nutrient consumption were computed by multiplying the frequency of consumption of each unit of food from the FFQ by the VD and nutrient content of the specified portion size. Composition values for dietary VD and other nutrients were estimated by means of a comprehensive Mexican database of food composition [32]. Information about use of specific brand and type of multivitamins was collected by asking current users about weekly number of multivitamins taken. Total VD represented the sum of VD intake from dietary and supplemental sources. Each nutrient was adjusted for total energy using the residual method [33]. Finally, in order to compute energy consumption, the daily frequency of consumption of each food was multiplied by the food’s energy content.

### Anthropometric assessment

As we reported previously [27–30], participants’ weight and height were measured by trained nurses using standardized procedures (reproducibility analysis showed concordance coefficients from 0.83 to 0.90). Weight (kg) and height squared (m^2^) were used to calculate body mass index (BMI kg/m^2^). Waist circumference was measured at the high point of the iliac crest at the end of normal expiration, to the nearest 0.1 cm, with a steel measuring tape, which was placed below any clothing, directly touching the participants’ skin. In the present study, being overweight or obese was defined as BMI ≥ 25 kg/m^2^ [34]. Abdominal obesity was defined as a waist circumference of >102 cm in men and >88 cm in women [35].

### Biomarkers assessment

A fasting venous blood sample (fasting time was 12 hours) was collected from each participant. Plasma triglycerides were measured with a colorimetric method following enzymatic hydrolysis performed with the lipase technique. High-density lipoprotein cholesterol (HDL-c) was measured by clearance method; in this method non HDL-c lipoprotein is removed in the first step of the reaction (clearance step). Low-density lipoprotein cholesterol (LDL-c) was measured by clearance method; finally, total cholesterol was measured by colorimetric method following enzymatic assay. All biomedical assays were performed using a Selectra XL instrument (Randox).

### High lipid profile assessment

High lipid profile was defined according to the criteria put forth in the report of the National Cholesterol Education Program ATP-III, that defines a high lipid profile as: high serum triglycerides ≥150 mg/dL, high serum total cholesterol ≥200 mg/dL, high LDL-c ≥100 mg/dL, and low HDL-c ≤40 mg/dL in men and ≤50 mg/dL in women [35].

### Cardiovascular disease risk assessment

Cardiovascular disease (CVD) risk was calculated using a recalibration of the Framingham coronary heart disease prediction scores [36, 37]. We first estimated the predicted risk of total CVD, applying the β-coefficients of Cox proportional hazards model obtained from the Framingham population by Wilson et al. [36], which included age, current smoking, type 2 diabetes, blood pressure regardless of hypertension treatment (predefined BP categories), serum LDL-c (predefined categories), and HDL-c (predefined categories). We used this equation to calculate each participant’s 10-year predicted probability of CVD.

The main outcome of the current analysis was the development of more than 10 percent risk of CVD in ten years. We defined participants as at low CVD risk when they had less than 10 percent risk in ten years. Subjects who had more than 10 percent risk in ten years were defining has having a CVD risk (moderate/elevated) by the American Heart Association standards [38].

### Non-dietary variables assessment

Demographic characteristics (e.g., age, sex, and education), medical history, and lifestyle, including alcohol and tobacco consumption, were collected by means of self-administered questionnaire. Physical activity was assessed using International Physical Activity Questionnaire (IPAQ). Participants were asked about their daily recreational activity, leisure activity, daily activity and any physical labor associated with employment. Participants reported the time they spent each week on activities such as running and walking during a typical week in the previous year. Each activity was given a value in metabolic equivalent tasks (METs) and total METs per week was computed [28–30].

### Statistical analysis

Descriptive analyses of the main variables of interest (including age, BMI, waist circumference, physical activity, and total energy intake, etc.) across quintiles of total vitamin D intake were performed. Analysis of variance (ANOVA) was used to evaluate mean differences across quintiles of total dietary VD consumption for continuous variables. The chi-square test was used to determine differences in the distribution of categorical variables across VD quintiles.

The influence of the total dietary VD intake on lipid profile, BMI, abdominal obesity, and scores of CVD risk was evaluated using multivariate lineal regression models in which these variables were analyzed as a continuous.

Finally, to estimate the magnitude of the association between specific categories of total dietary VD intake and abnormal lipids profile, obesity, abdominal obesity, as well as elevated CVD risk, we computed adjusted odds ratios (OR) and 95% confidence intervals (95% CI) using multiple logistic regression models.

To assess possible effect modification, we explored analyses stratified by body mass index (two categories: <25 kg/m^2^ vs ≥ 25 kg/m^2^). We tested the significance of the interaction with a likelihood ratio test by comparing a model with the main effects of each intake and the stratifying variable and the interaction terms with a reduced model with only the main effects.

All *P* values presented are two sided; *P* < 0.05 was considered statistically significant. The statistical analyses were performed using the STATA statistical software package, version 13.0 (Stata Corp. LP: College Station, TX).

## Results

Participants’ baseline characteristics (age, sex, body composition, clinical parameters, and dietary information) are shown in Table 1. A total of 6294 subjects (1820 men and 4474 women) with a mean age of 42 years, were included in the final analysis. Of these, the corresponding median intakes in the lowest and highest quintiles were 70.4 and 516.2 international units (IU) of vitamin D_3_ (cholecalciferol) respectively. A higher VD intake was observed in subjects with lower BMI, fewer prevalence of overweight and obesity and abdominal obesity, increased physical activity levels, decreased use of tobacco, and increased use of multivitamins (*P* for trend <0.001) were observed across increasing VD quintiles. In addition, subjects in the highest quintile of VD intake had a lower prevalence of diabetes and lipid abnormalities compared with subjects in the lowest quintile of VD consumption. Finally, data shows that subjects in the highest quintile of VD intake have a lower likelihood of > 10% risk of 10-year cardiovascular disease than those in the lowest quintile.

**Table 1:** Baseline characteristics by categories of total vitamin D intake in the Health Workers Cohort Study.

Characteristics	Quintiles of vitamin D intake										
	Q1		Q2		Q3		Q4		Q5		
	(n = 1254)		(n = 1261)		(n = 1256)		(n = 1264)		(n = 1259)		
	Mean	SD	Mean	SD	Mean	SD	Mean	SD	Mean	SD	P value**
Age, years	42.9	12.9	41.7	12.9	41.9	12.9	41.9	13.1	42.6	14.3	0.112
Women, %	68.1		70.2		72.9		72.6		71.6		0.047
BMI, kg/m^2^	26.7	4.4	26.5	4.2	26.4	4.2	26.2	4.4	26.0	4.4	0.002
Overweight and obesity, %* ^a^	62.0		61.9		60.0		56.4		55.3		<0.001
Abdominal Obesity, %* ^b^	43.5		41.6		41.9		40.4		39.4		0.030
Diabetes, %* ^c^	5.4		4.3		4.8		4.6		4.7		0.757
Hypercholesterolemia, %* ^d^	37.6		38.7		38.7		37.4		37.9		0.799
Hypoalphalipoproteinemia, %* ^e^	79.0		75.6		77.7		75.5		75.3		0.040
High LDL-c, %*^f^	64.9		66.2		64.9		66.5		64.6		0.961
Hypertriglyceridemia, %*^g^	45.2		40.6		40.1		38.3		37.5		<0.001
Vitamin suplements, %*	30.2		32.1		33.8		35.6		38.9		<0.001
Current smoker, %*	23.8		20.5		21.3		18.8		14.5		<0.001
Physical activity, mets/week	140.7	62.6	146.8	64.8	148.5	63.3	154.6	65.2	154.7	64.8	<0.001
10-year cardiovascular risk score^h^	5.6	6.4	5.1	5.7	5.1	6.1	5.0	6.2	4.8	5.9	0.02
Medium/High 10-year cardiovascular diseas, %^i^	15.5		14.8		14.8		14.0		13.5		0.05
Total energy intake, kcal/day	1642	666	1868	683	2098	725	2415	830	3043	1071	<0.001
Vitamin D intake (IU/day)	70.4	25.3	140.8	17.7	203.4	20.2	295.0	34.2	516.2	34.2	<0.001
Sodium, mg/day	1477	834	1669	731	1917	783	2238	911	2883	1244	<0.001
Fiber, g/day	14.0	9.3	15.0	8.9	16.5	8.7	18.6	10.2	22.2	13.2	<0.001
Saturated fats, g/day	13.7	7.2	17.2	7.0	20.6	7.5	24.7	9.5	34.7	16.0	<0.001
Polyunsaturated fats, g/day	8.2	4.9	9.2	4.9	10.1	4.8	11.3	5.4	13.8	6.9	<0.001
Magnesium, mg/day	275.1	117.0	317.8	133.1	357.3	134.2	417.1	152.3	534.5	206.2	<0.001

* Data are presented as percentage.

** ANOVA test was used for quantitative variables; χ2 test was used for qualitative variables.

^a^ Overweight and obesity; defined as BMI ≥ 25K/m^2^.

^b^ Abdominal obesity; defined as waist circumference ≥88cm for women and ≥102 for men.

^c^ Diabetes; defined as fasting plasma glucose ≥ 126.0 mg/dL.

^d^ Hypercholesterolemia; defined as plasma total cholesterol ≥ 200 mg/dL.

^e^ Hypoalphalipoproteinemia; defined as plasma HDL-c ≤ 50.0 mg/dL for women, ≤ 40.0 mg/dL for men.

^f^ High LDL-c; defined as plasma LDL-c ≥ 130.0 mg/dL.

^g^ Hypertriglyceridemia; defined as plasma triglycerides ≥ 150 mg/dL.

^h^ The Cardiovascular risk score was computed using the Framingham risk score

^i^ Medium/High cardiovascular disease risk; defined as >10%.

The effect of VD on cardiovascular risk markers is shown on Table 2. After adjusting for age, sex, multivitamin use, BMI, physical activity, alcohol, saturated fats, polyunsaturated fats, fiber, energy, glycemic load, smoking status, postmenopausal hormone use, place of residence and season we observed that subjects in the highest quintile of VD intake presented a decrease of 14.6 mg/dL in triglycerides (*P* for trend = 0.01); 0.9 kg/m^2^ in BMI (*P* for trend <0.001); 2.0 cm in waist circumference (*P* for trend = 0.001) and 0.8 points in the Framingham cardiovascular risk score (*P* for trend = 0.002) compared with the subjects in the lower VD category.

**Table 2:** Multivariate regression model for evaluating the association between vitamin D intake and cardiovascular risk markers in a Mexican adult population.

Variables	Quintiles of vitamin D intake									
	Q1	Q2		Q3		Q4		Q5		
	β	β	(95% CI)	β	(95% CI)	β	(95% CI)	β	(95% CI)	*P* for trend*
Total cholesterol, mg/dL^a^	0.0	2.2	(-0.7, 5.2)	1.8	(-1.2, 4.9)	2.9	(-0.5, 6.5)	2.6	(-0.4, 5.9)	0.101
HDL Cholesterol, mg/dL^a^	0.0	1.1	(0.2, 2.0)	1.3	(0.3, 2.2)	1.7	(0.6, 2.6)	2.8	(1.6, 4.1)	<0.001
LDL Cholesterol, mg/dL^a^	0.0	1.9	(-1.1, 5.0)	1.5	(-1.5, 4.5)	2.1	(-0.9, 5.7)	2.7	(-0.6, 6.1)	0.097
Triglycerides, mg/dL^a^	0.0	-8.0	(-15.7, -2.2)	-9.9	(-17.8, -1.8)	-11.1	(-19.1, -4.6)	-14.6	(-24.3, -4.9)	0.01
Body mass index, kg/m^2^^b^	0.0	-0.07	(-0.4, 0.2)	-0.2	(-0.6, 0.1)	-0.5	(-0.8, -0.1)	-0.9	(-1.2, -0.3)	<0.001
Waist circumference, cm^b^	0.0	-0.7	(-1.6, 0.2)	-0.8	(-1.8, 0.8)	-1.4	(-2.4, -0.4)	-2.0	0 (-3.1, -0.9)	0.001
Cardiovascular disease risk^c^	0.0	-0.3	(-0.8, 0.1)	-0.3	(-0.7, 0.3)	-0.5	(-0.8, 0.2)	-0.8	(-1.4, -0.3)	0.002

*Test for linear trend.

^a^ Model adjusted for age (years), sex, multivitamin use (yes or not), BMI (continuous), physical activity (quintiles), alcohol, saturated and polyunsaturated fats (quintiles), fiber (quintiles), energy (quintiles), glycemic load (quintiles), current smoking (never, past or current), postmenopausal hormone use (yes or not).

^b^ Model adjusted for age (years), sex, multivitamin use (yes or not), physical activity (quintiles), alcohol intake (quintiles), saturated and polyunsaturated fats (quintiles), fiber (quintiles), energy (quintiles), glycemic load (quintiles), current smoking (never, past or current), postmenopausal hormone use (yes or not).

^c^ Model adjusted for age (y), sex, multivitamin use (yes or not), BMI (continuous), physical activity (quintiles), alcohol (quintiles), saturated and polyunsaturated fats (quintiles), fiber, energy (quintiles), glycemic load (quintiles), place of residence, season, postmenopausal hormone use (yes or not) and history of myocardial infarction (yes or not).

Table 3 shows the ORs and 95% CIs of a multiple logistic regression analysis evaluating the association between VD intake, CVD, obesity, abdominal obesity and cardiometabolic risk factors. After adjustment for lifestyle and dietary covariates, total VD intake was inversely associated with some cardiometabolic risk factors such as low HDL-c, hypertrigliceridemia, obesity and abdominal obesity. For example: subjects in the higher category of VD intake had lower odds of presenting low HDL-c (OR = 0.65; 95%CI: 0.52, 0.82), hypertriglyceridemia (OR = 0.72; 95%CI: 0.59, 0.88), obesity (OR = 0.74; 95%CI: 0.61, 0.90), and abdominal obesity (OR = 0.74; 95%CI: 0.60, 0.91). Additionally, participants in the highest quintile of VD consumption were less likely to develop elevated 10-year CVD risk, compared with those in the lowest quintile (OR = 0.51; 95%CI: 0.33, 0.77; *P* for trend = 0.007).

**Table 3:** Multivariate logistic regression model for evaluating the association between vitamin D intake and cardiovascular risk markers in a Mexican adult population.

Variables	Quintiles of vitamin D intake									
	Q1	Q2		Q3		Q4		Q5		
	OR	OR	(95% CI)	OR	(95% CI)	OR	(95% CI)	OR	(95% CI)	*P* for trend*
Hypercholesterolemia^a^	1.0	1.05	(0.89, 1.24)	1.12	(0.93, 1.34)	1.17	(0.96, 1.40)	1.15	(0.92, 1.43)	0.174
Hypoalphalipoproteinemia ^a^	1.0	0.80	(0.69, 0.96)	0.83	(0.68, 1.03)	0.76	(0.64, 0.93)	0.65	(0.52, 0.82)	<0.001
Elevated LDL-c^a^	1.0	1.07	(0.92, 1.21)	0.99	(0.82, 1.18)	1.09	(0.92, 1.29)	0.93	(0.74, 1.15)	0.682
Hypertriglyceridemia^a^	1.0	0.83	(0.70, 0.98)	0.87	(0.73, 1.03)	0.77	(0.64, 0.92)	0.72	(0.59, 0.88)	0.006
Overweight and obesity^b^	1.0	0.99	(0.85, 1.19)	0.94	(0.80, 1.13)	0.79	(0.63, 0.92)	0.74	(0.61, 0.90)	<0.001
Abdominal obesity^b^	1.0	0.91	(0.76, 1.09)	0.93	(0.77, 1.12)	0.82	(0.67, 0.99)	0.74	(0.60, 0.91)	0.007
Medium/high predicted cardiovascular disease risk^c^	1.0	0.93	(0.72, 1.26)	0.87	(0.61, 1.22)	0.69	(0.53, 1.09)	0.51	(0.33, 0.77)	0.007

*To assess the overall trend of OR across increasing quintile of dietary pattern scores we computed the Mantel-Haenszel extension chisquare test.

^a^ Model adjusted for age (years), sex, multivitamin use (yes or not), BMI (continuous), physical activity (quintiles), alcohol, saturated and polyunsaturated fats (quintiles), fiber (quintiles), energy (quintiles), glycemic load (quintiles), current smoking (never, past or current), postmenopausal hormone use (yes or not).

^b^ Model adjusted for age (years), sex, multivitamin use (yes or not), physical activity (quintiles), alcohol intake (quintiles), saturated and polyunsaturated fats (quintiles), fiber (quintiles), energy (quintiles), glycemic load (quintiles), current smoking (never, past or current), postmenopausal hormone use (yes or not).

^c^ Model adjusted for age (y), sex, multivitamin use (yes or not), BMI (continuous), physical activity (quintiles), alcohol (quintiles), saturated and polyunsaturated fats (quintiles), fiber, energy (quintiles), glycemic load (quintiles), place of residence, season, postmenopausal hormone use (yes or not) and history of myocardial infarction (yes or not).

**Fig 1:**
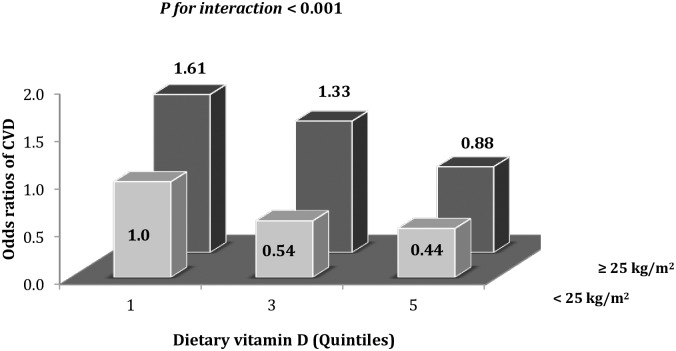
Joint association of dietary vitamin D (quintiles) and body mass index (<25 kg/m^2^ and ≥ 25 kg/m^2^) with the predicted cardiovascular disease risk. Reference group for comparisons were subjects in lowest quintile of vitamin D intake with body mass index < 25 kg/m^2^. Odds ratio were adjusted for: age (years), sex, multivitamin use (yes or not), BMI (continuous), physical activity (quintiles), alcohol (quintiles), saturated and polyunsaturated fats (quintiles), fiber, energy (quintiles), glycemic load (quintiles), place of residence, season, postmenopausal hormone use (yes or not) and history of myocardial infarction (yes or not).

We examined the joint effect of VD intake and body mass index by cross classifying the study population by both variables. The odds ratio from these stratified analyses was 1.61 (95% CI: 1.08–2.57; *P* for interaction <0.001) when subjects with low VD consumption and overweight/obesity were compared with subjects with a low consumption of VD and body mass index < 25 kg/m^2^ (Fig 1).

## Discussion

To the best of our knowledge, this is the first study assessing the association between VD intake and CVD risk in Mexican adult population. We found that our study population on average consumed less VD than the recommended daily allowance [39]. As we reported in a previous study, approximately 96% of men and women had inadequate intake of VD [40]. These findings are similar to those observed in another adult population [41, 42]. For example, Bailey et al. [41], found that approximately 95% of women and 96% of men in the US had inadequate consumption of VD. The inadequate consumption of VD in the diet is explained easily since VD is found in only few foods and in low amounts.

In literature, no studies have investigated the association between dietary VD and cardiovascular disease risk factors. Despite the fact, in a set of cross-sectional studies [43–47], the relationship between VD and cardiovascular disease risk factors have been evaluated considering serum levels of VD. In our population, the average blood plasma concentrations of triglycerides, waist circumference, and BMI decreased across the quartiles of VD intake. In addition, we found that blood plasma concentrations of HDL-c increased as dietary VD intake increased. As we noted above, most cross-sectional studies [43–49], high serum VD have been related to lower triglyceride blood plasma concentrations [40–45], and higher HDL-c levels [43–48]. Furthermore, our analyses shows that subjects in the highest quintile of VD intake would entail on average 14.6 mg/dL deficit of triglycerides and a 2.8 mg/dL excess of HDL-c, compared to the average levels of our study population. Similar to our results Ramly and coworkers, in a clinical trial [50], found that subjects who receiving VD had on average 11.2 mg/dL lower concentrations of triglycerides and 0.8 mg/dL higher concentrations of HDL-c compared with subjects who did not receive VD. Additionally, our results suggest that subjects in the highest quintile of VD intake had on average 0.9 lower units (kg/m^2^) of BMI, and 2.0 cm in waist circumference. In accordance with this result, other studies [43–45] have reported that higher serum levels of VD are associated with lower waist circumference (on average between 0.4 to 3.2 cm), and lower BMI [46].

In our study, logistic regression analysis shows that subjects in the higher dietary VD group have 28% lower odds of hypertriglyceridemia (*P* for trend = 0.006), 35% lower odds of low HDL-c, and 26% lower odds of being obese or abdominal obese than subjects in the lowest quintile of dietary VD intake. However, serum total cholesterol and LDL-c concentrations were not related to VD intakes. Compared to our results, Martins et al. [51] found that subjects in the lowest quartile of serum VD levels had higher odds of hypertriglyceridemia (OR = 1.47; 95%CI: 1.30, 1.65), higher odds of obesity (OR = 2.29; 95%CI: 1.99, 2.63).

Our data also suggests an inverse relationship between VD intake and 10-year CVD risk. We observed that subjects in the highest quintile had 49% lower odds of 10-year CVD risk (OR = 0.51; 95% CI:0.33, 0.77; *P* for trend = 0.007). This result is consistent with a study that found that subjects in the higher group of total VD intake was associated with a decreased risk of CVD; the relative risks (95% CIs) for a comparison of participants who met the Dietary Reference Intake of VD (≥ 600 IU/day) with participants whose VD intake was <100 IU/day was 0.84 (95%CI: 0.72, 0.97; *P* for trend = 0.009) for men and 1.02 (95%CI: 0.89, 1.17; *P* for trend = 0.12) for women [52]. In addition, other study conducted by Wang et al. [53] reported that subjects in the lowest category of serum VD had 81% higher risk of CVD (RR = 1.81; 95%CI: 1.03, 3.18), compared with subjects in the highest category.

Several mechanisms support the possible beneficial effects of VD on cardiovascular disease [53, 54]. First, this may be explained by the large number of receptors for VD, which are present in many cells of the cardiovascular system. Experimental evidence indicates that the active form of VD (1,25 (OH) D) inhibits the expression of the gene for renin, regulating in this way the system renin-angiotensin which results in the modulation of the growth and proliferation of vascular cells and cardiomyocytes, as well as processes of inflammation and thrombosis [54]. Vascular and endothelial cells express receptors for VD and have the ability to convert 25 (OH) D circulating in (1,25 (OH) D). On the other hand, vitamin D deficiency along with secondary hyperparathyroidism promote hypertrophy of cardiomyocytes and vascular remodeling; studies suggest that PTH has a inflammatory effect, since it stimulates the release of cytokines [54]. In a study published in 2008, Wang [53] points out the possible interaction between VD and hypertension, and some experimental studies suggest that VD deficiency promotes the development of hypertension through the mechanism of the renin angiotensin system before mentioned. In this regard, some clinical trials have discussed associations of low VD concentrations with high blood pressure, coronary artery, calcification and cardiovascular diseases such as heart attack myocardial infarction, congestive failure [54, 55].

Some limitations of the present study must be highlighted. As we stated in previous studies [28,30], the participants in this cohort study are adults from a specific segment of the Mexican population: working class, seemingly healthy individuals. While they cannot be considered representative of the Mexican adult population as a whole, they may be considered representative of middle to low income adults residing in the urban areas of central Mexico. Other limitations are related to the measurement of nutrient consumption and effects. First, VD intake was assessed from a single measurement of FFQ, which is subject to random error that would tend to underestimate the true association between VD intake and the cardiovascular disease risk in our study. However, these errors are unlikely to affect our results, since the FFQ that we used in this study has been previously validated [31] as reasonably reflecting long-term dietary intake. Second, we did not separately compare the effects of dietary VD and supplementary VD, but since subjects’ intake of VD from supplements was minor compared with dietary intake, this was not likely to have influenced the results. Third, there is a high degree of multicolinearity among VD and other nutrients that makes it difficult to completely separate the independent effects of VD from those of nutrients like calcium and retinol. At the respect, in the present study we evaluated the relationship between dietary calcium and retinol and the 10-year risk of CVD observed null associations (data not shown). Another important limitation is that we could not measure serum 25-hydroxyvitamin D levels; therefore, we could not evaluate the relationship between serum vitamin D and cardiovascular disease risk. While our analyses considered many potential covariates that might confound the observed associations; however, the possibility of residual confounding still remains. Additionally, given that this study was cross-sectional in nature, temporal associations between VD intake and cardiovascular disease risk cannot be determined. The major strength of the current study is the inclusion of data from a large sample size of middle-aged men and women.

In summary, our findings suggest that VD intake is associated with decreased odds of having 10-year risk of CVD. We also noted that higher VD intake reduced the odds of obesity and central obesity, as well as, the odds of low HDL-c. Additionally, the association of VD intake with 10-year CVD appeared to be mediated by BMI. Further observational research, particularly prospective studies, is needed to confirm our findings in Mexican adult population.

## Supporting Information

S1 File STROBE statement. Checklist of items that should be included in reports of cross-sectional studies.
(DOC)
